# Conversion from long-term cultivated wheat field to Jerusalem artichoke plantation changed soil fungal communities

**DOI:** 10.1038/srep41502

**Published:** 2017-01-30

**Authors:** Xingang Zhou, Jianhui Zhang, Danmei Gao, Huan Gao, Meiyu Guo, Li Li, Mengliang Zhao, Fengzhi Wu

**Affiliations:** 1 Department of Horticulture, Northeast Agricultural University, Harbin, China; 2 Key Laboratory of Biology and Genetic Improvement of Horticultural Crops (Northeast Region), Ministry of Agriculture, Harbin, China; 3 Institute of Horticulture, Qinghai Academy of Agriculture and Forestry Sciences, Xining, China

## Abstract

Understanding soil microbial communities in agroecosystems has the potential to contribute to the improvement of agricultural productivity and sustainability. Effects of conversion from long-term wheat plantation to Jerusalem artichoke (JA) plantation on soil fungal communities were determined by amplicon sequencing of total fungal ITS regions. Quantitative PCR and PCR-denaturing gradient gel electrophoresis were also used to analyze total fungal and *Trichoderma* spp. ITS regions and *Fusarium* spp. *Ef1α* genes. Results showed that soil organic carbon was higher in the first cropping of JA and Olsen P was lower in the third cropping of JA. Plantation conversion changed soil total fungal and *Fusarium* but not *Trichoderma* spp. community structures and compositions. The third cropping of JA had the lowest total fungal community diversity and *Fusarium* spp. community abundance, but had the highest total fungal and *Trichoderma* spp. community abundances. The relative abundances of potential fungal pathogens of wheat were higher in the wheat field. Fungal taxa with plant growth promoting, plant pathogen or insect antagonistic potentials were enriched in the first and second cropping of JA. Overall, short-term conversion from wheat to JA plantation changed soil fungal communities, which is related to changes in soil organic carbon and Olsen P contents.

Jerusalem artichoke (JA) (*Helianthus tuberosus* L.) is an economically important crop, which can be used as a human food and livestock feed, and as a source of inulin (as sweetener or for ethanol production)^1^,^2^. JA is also easily grown in coastal and semiarid areas because of its high drought and salinity tolerance^3^. The economic benefit of JA is higher than grain crops; therefore, large areas of field used for main food production, for example wheat (*Triticum aestivum* L.), have been converted to JA plantation in China^4^,^5^.

As a major component of global biodiversity, soil microorganisms play pivotal roles in terrestrial ecosystem processes, including soil formation, carbon and nitrogen cycling, and plant nutrient acquisition^6^,^7^. Plant litter and root exudates provide important carbon resources for soil microorganisms and changes in these plant-derived organic matters can affect soil microbial communities^8^,^9^. Moreover, plant root exudates and litter chemistry differ among species and have different effects on below-ground ecosystem processes^8^,^10^,^11^. Therefore, changes in cultivated crop species can affect soil microbial community activity, composition and function^12^,^13^. Knowledge about how land use conversion influences soil microbial communities is helpful for understanding how agricultural practices influence soil processes which are mediated by microorganisms and developing cultivation practices to increase crop growth and health through manipulating soil microbial communities^14^.

Soil fungi represent an essential functional component of soil microbial communities as decomposers, mutualists and pathogens, and can affect plant growth and health in agroecosystems^6^,^15^. *Fusarium* (Sordariomycetes: Hypocreales: Nectriaceae) and *Trichoderma* (teleomorph *Hypocrea* spp., Sordariomycetes: Hypocreales: Hypocreaceae) spp. are diverse genera of filamentous ascomycete fungi that contain many species of agricultural importance^16^,^17^. Beside playing important roles in organic matter decomposition, many species in *Fusarium* spp. are phytopathogenic fungi, which can infect a wide range of crop plants and cause wilting diseases^18^,^19^. *Trichoderma* spp. are opportunistic, avirulent plant symbionts, as well as being parasites of other fungi, and can exert beneficial effects on plants through inhibiting plant pathogens and promoting plant growth^20^.

In this study, the effects of conversion from long-term cultivated wheat field to JA plantation on soil total fungal, *Trichoderma* and *Fusarium* spp. communities were analyzed. In the open field, wheat was successively planted for more than 20 years and then converted to cultivate JA for three years from 2010 to 2012, respectively. Soil fungal community abundance, structure and composition were analyzed by quantitative PCR (qPCR), PCR-denaturing gradient gel electrophoresis (PCR-DGGE), and Illumina Miseq sequencing. We hypothesize that soil microbial communities differ between wheat and JA fields since land use conversion can induce changes in plant-derived carbon resources. Plants have species-specific effects on soil microbial communities, which may strengthen over time^21^; and successive monocropping can adversely affect soil biodiversity^22^,^23^,^24^. Thus, we also hypothesize that soil microbial community structure change and its diversity decrease after repeated cultivation of JA.

## Results

In this study, triplicate bulk soil samples were taken from a long-term cultivated wheat field and fields converted to JA plantation for one, two and three years, respectively. Then, we analyzed the soil chemical properties and soil fungal communities. Soil total fungal, *Trichoderma* and *Fusarium* spp. community diversities and abundances were determined by analyzing total fungal and *Trichoderma* spp. ITS regions and *Fusarium* spp. *Ef1α* gene.

### Illumina Miseq sequencing data

Illumina Miseq sequencing generated 469,826 high quality fungal sequences with an average read length of 263 bp, and the number of sequences per sample ranged from 34,468 to 44,657 (Table 1). A total of 1,073 operational taxonomic units (OTUs) were identified at 97% similarity. The Good’s coverage, which reflects the captured diversity, was larger than 99% for all samples. The rarefaction curves and Shannon-Wiener curves also indicated that the sequencing data represented most of the total fungal community composition (Fig. S1).

### Fungal community composition

**Figure 1:**
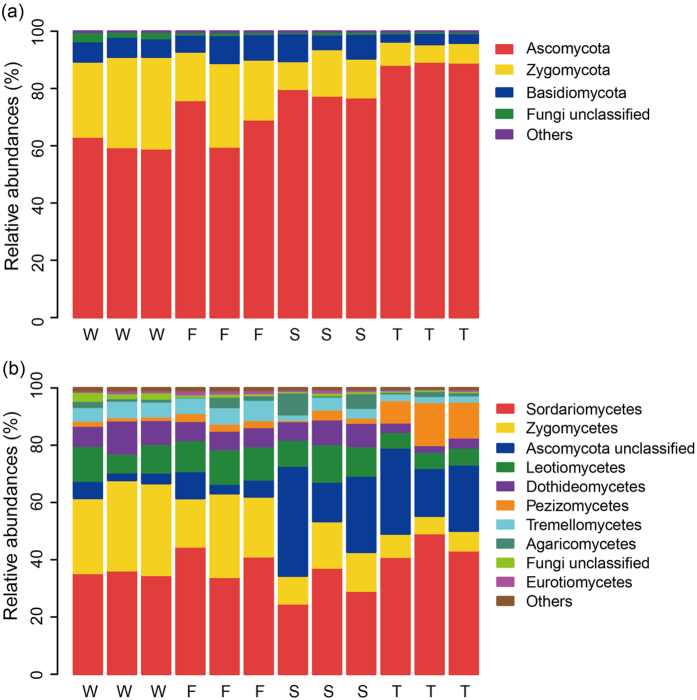
Relative abundances of main soil fungal phyla (**a**) and classes (**b**) in the wheat field (W), the first (F), second (S) and third (T) cropping of Jerusalem artichoke.

Across all samples, five fungal phyla were detected. Ascomycota, Basidiomycota and Zygomycota were the dominant phyla, which accounted for more than 98% of the sequences (Fig. 1a). Chytridiomycota and Glomeromycota were also detected at relatively low abundances (relative abundance < 1%). Compared with the wheat field, the relative abundance of Ascomycota was higher (F = 24.26, P = 0.0002) while the relative abundance of Zygomycota was lower in the second and third cropping of JA (F = 19.87, P = 0.0005) (Figs 1a and S2). The relative abundance of Basidiomycota was the lowest in the third cropping of JA (F = 5.76, P = 0.0213).

At the class level, all samples were dominated by Sordariomycetes, Leotiomycetes, Dothideomycetes, Pezizomycetes, Tremellomycetes and Agaricomycetes (Fig. 1b). The wheat field had higher relative abundances of Zygomycetes and Microbotryomycetes than the second and third cropping of JA (F = 19.87, 9.91; P = 0.0005, 0.0045; respectively) (Fig. 1b and S2). The first cropping of JA had the highest relative abundance of Saccharomycetes (F = 15.38, P = 0.0011) (Fig. S2). The third cropping of JA had a higher relative abundance of Pezizomycetes (F = 18.68, P = 0.0006) but lower relative abundances of Zygomycetes, Leotiomycetes and Dothideomycetes (F = 19.87, 6.40, 11.05; P = 0.0005, 0.0161, 0.0032; respectively) (Figs 1b and S2).

**Figure 2:**
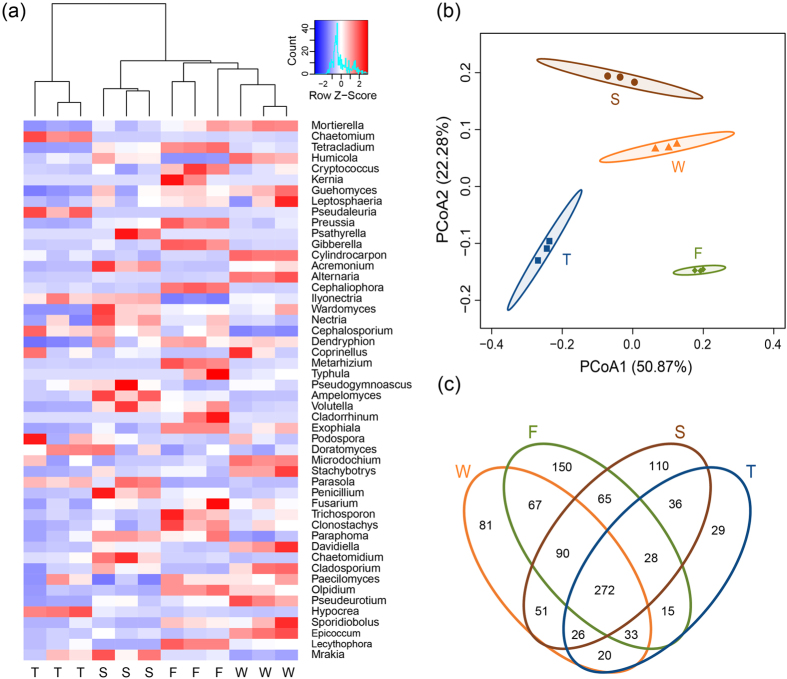
Heat map (**a**), PCoA (**b**) and Venn diagram (**c**) analyses of soil fungal communities in the wheat field (W), the first (F), second (S) and third (T) cropping of Jerusalem artichoke. In the heat map (**a**), the relative abundances of the top 50 most abundant classified fungal genera were identified in each sample by colors deduced from the raw Z-scores. Hierarchical clustering of soil samples was performed using average clustering method with the Euclidean distances. The PCoA plot (**b**) was based on the weighted UniFrac distance at the OTU level (97% sequence similarity) of fungal communities. Ellipses indicate 95% confidence interval for replicates. Venn diagram (**c**) demonstrated the numbers of shared and unique observed OTUs at 97% similarity among treatments.

At the genus level, more than 260 genera were detected. *Mortierella* and *Chaetomium* spp. were dominant genera, which had mean relative abundances of 18.09% and 7.42%, respectively (Table S1). The wheat field had higher relative abundances of *Mortierella, Cylindrocarpon, Alternaria, Microdochium, Stachybotrys, Davidiella, Pseudeurotium* and *Epicoccum* spp. (F = 19.89, 108.50, 190.73, 13.02, 15.94, 18.93, 27.99, 45.28; P = 0.0005, < 0.0001, < 0.0001, 0.0019, 0.0010, 0.0005, 0.0001, < 0.0001; respectively) (Fig. 2a, Table S1). The first cropping of JA had higher relative abundances of *Tetracladium, Cryptococcus, Preussia, Gibberella, Cephaliophora, Metarhizium, Exophiala, Trichosporon, Clonostachys, Olpidium* and *Lecythophora* spp. (F = 140.60, 10.12, 103.93, 87.36, 346.21, 151.03, 19.70, 9.38, 12.22, 54.16, 71.35; P =< 0.0001, 0.0042, <0.0001, <0.0001, <0.0001, <0.0001, 0.0005, 0.0054, 0.0023, <0.0001, <0.0001; respectively). The second cropping of JA had higher relative abundances of *Acremonium, Ampelomyces, Penicillium* and *Chaetomidium* spp. (F = 20.03, 19.21, 9.43, 12.17; P = 0.0004, 0.0005, 0.0053, 0.0024; respectively). The third cropping of JA had higher relative abundances of *Chaetomium, Pseudaleuria* and *Hypocrea* spp. (F = 83.27, 32.60, 160.07; P < 0.0001, < 0.0001, < 0.0001; respectively).

### Fungal community diversity and structure

The number of observed OTUs, Chao1, ACE, and Shannon indices were similar in samples from the wheat field, the first and second cropping of JA, but were significantly lower in the third cropping of JA (F = 23.47, 9.97, 11.75, 13.68; P = 0.0003, 0.0044, 0.0027, 0.0016; respectively) (Table 1). The Simpson index was significantly higher in the third cropping of JA than in the wheat field and the first cropping of JA (F = 6.39, P = 0.0161).

Principal coordinates analysis (PCoA) analysis at the OTU level showed that samples from the wheat field, the first, second and third cropping of JA were separated from each other (Fig. 2b). Analysis of similarities (ANOSIM), non-parametric multivariate ANOVA (adonis), and multiple response permutation procedure (MRPP) analyses demonstrated that soil fungal community structure differed among soil samples with different plantation history (P < 0.001) (Table S2).

### Shared and unique OTUs

Venn diagram analysis of OTUs at 97% sequence similarity showed that all samples shared 272 OTUs, which accounted for 25.35% of the total OTUs observed (Fig. 2c). At the class level, these shared OTUs were mainly composed of sequences belonging to Sordariomycetes, unclassified Ascomycota and Zygomycetes. Samples from the wheat field had 81 unique OTUs, which were mainly composed of sequences belonging to unclassified Fungi and unclassified Ascomycota (Table S3). At the genus level, these OTUs were dominated by sequences belonging to *Lachnella* spp. (4.35%) (data not shown).

Among all samples, the first cropping of JA had the highest number of unique OTUs (150) and the third cropping of JA had the lowest number of unique OTUs (29) (Fig. 2c). OTUs unique to the first cropping of JA were dominated by sequences belonging to Sordariomycetes, Zygomycetes, Leotiomycetes, unclassified Fungi, and Agaricomycetes at the class level (Table S3), and *Mortierella* (15.88%) and *Scytalidium* spp. (6.56%) at the genus level. OTUs unique to the second cropping of JA were mainly composed of sequences belonging to *Mortierella* (13.50%) and *Powellomyces* spp. (7.56%). OTUs unique to the third cropping of JA were dominated by sequences belonging to Pezizomycetes, Sordariomycetes and Dothideomycetes with *Preussia* spp. (48.02%) as the dominate genus.

### *Fusarium* and *Trichoderma* community compositions

Miseq sequencing generated five OTUs that classified as *Fusarium* spp. and one OTU as *Trichoderma/Hypocrea* sp. (Table S4). However, none of these OTUs could be aligned at the species level. For *Fusarium* spp., four OTUs were detected in the first cropping of JA, while only two OTUs (OTU721 and OTU504) were detected in the wheat field and the third cropping of JA. Compared with the wheat field and the second cropping of JA, the third cropping of JA had significantly lower number of sequences in OTU504 (F = 7.28, P = 0.0113). For *Trichoderma/Hypocrea* sp., OTU703 was detected in all samples and the number of its sequences was the highest in the third cropping of JA (F = 149.06, P < 0.0001).

### Fungal community structures as revealed by PCR-DGGE

DGGE profiles of the total fungal and *Fusarium* spp. communites differed among soil samples from the wheat field, the first, second and third cropping of JA with respect to the number and position of bands (Fig. S3a,b). For total fungal community, Shannon diversity and Evenness indices did not significantly differ among samples (Table S5). For the *Fusarium* spp. community, Shannon diversity and Evenness indices were higher in the wheat field and the first cropping of JA than in the second and third cropping of JA (F = 200.79, 166.96; P < 0.0001, <0.0001; respectively). For both total fungal and *Fusarium* spp. communities, soil from the third cropping of JA had the lowest number of visible bands (F = 9.38, 251.00; P = 0.0054, <0.0001; respectively).

**Figure 3:**
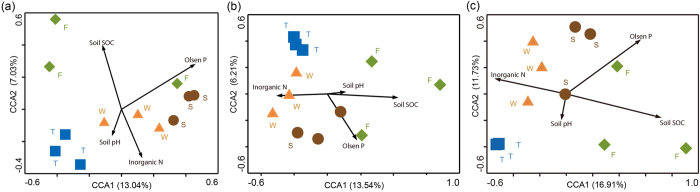
CCA analysis of soil fungal (**a**), *Fusarium* spp. (**b**) community structures based on PCR-DGGE and soil fungal community structure based on Miseq sequencing (**c**) with soil chemical properties. W, F, S and T indicate soil samples from the wheat field, the first, second and third cropping of Jerusalem artichoke, respectively.

The canonical correspondence analysis (CCA) of total fungal and *Fusarium* spp. community DGGE profiles showed that, except for the first cropping of JA, samples with differing plantation history could be separated from each other (Fig. 3a,b). ANOSIM, adonis and MRPP analyses showed that soil total fungal and *Fusarium* spp. community structures were significantly influenced by plantation conversion from wheat field to JA (P < 0.001) (Table S2).

For the *Trichoderma* spp. community, the banding patterns of DGGE profile were similar among samples (Fig. S3c). Diversity indices calculated from the DGGE profile also did not differ among samples (data not shown).

### Fungal community abundances

**Figure 4:**
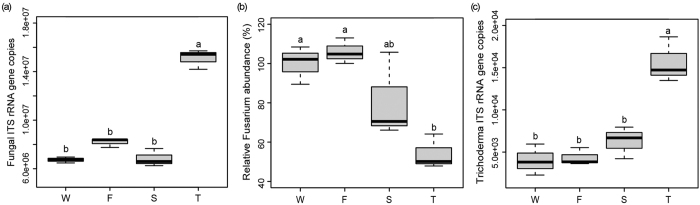
Soil total fungal (**a**), *Fusarium* (**b**) and *Trichoderma* (**c**) spp. community in the wheat field (W), the first (F), second (S) and third (T) cropping of Jerusalem artichoke as determined by quantitative PCR. Values (mean ± SE) with different letters are significantly different at the 0.05 probability level (Tukey’s HSD test).

qPCR analysis showed that conversion from wheat to JA plantation significantly influenced the total fungal, *Fusarium* and *Trichoderma* spp. community abundances (F = 136.49, 9.49, 23.33; P < 0.0001, =0.0052, 0.0003; respectively) (Fig. 4). The wheat field, the first and second cropping of JA had similar soil total fungal, *Fusarium* and *Trichoderma* spp. abundances. However, total fungal and *Trichoderma* spp. abundances were the highest in the third cropping of JA (F = 136.49, 23.33; P < 0.0001, =0.0003; respectively) (Fig. 4a,c). Compared with the wheat field and the first cropping of JA, the third cropping of JA had significantly lower relative abundance of *Fusarium* spp. community (F = 9.49, P = 0.0052) (Fig. 4b).

### Soil chemical properties and their relationships with soil fungal communities

Conversion from long-term cultivated wheat field to JA plantation did not change soil pH and inorganic N content (Table 2). The first cropping of JA had the highest soil organic carbon (SOC) content (F = 11.14, P = 0.0031) while the third cropping of JA had the lowest soil Olsen P (F = 12.68, P = 0.0021) (Table 2).

The relationships between soil fungal communities and soil chemical properties were assessed by CCA analysis and Mantel test. In the CCA plots of soil fungal community structures based on Miseq sequencing Fig. 3c and PCR-DGGE Fig. 3a, *Fusarium* spp. community based on PCR-DGGE, SOC and Olsen P were two longer arrows (Fig. 3b), which indicated that both SOC and Olsen P had strong effects on soil fungal and *Fusarium* spp. community structures. Mantel test showed that soil total fungal community structure, as analyzed by Illumina Miseq sequencing and PCR-DGGE, and *Fusarium* spp. community structure, as analyzed by PCR-DGGE, were significantly correlated to SOC and Olsen P (P < 0.05) but not to soil pH and inorganic N (Table S6).

## Discussion

Soil microbial communities are of fundamental importance to sustainable crop production by increasing the availability of mineral nutrients, producing phytohormones, inducing plant resistance to pathogens, and suppressing plant pathogens^6^,^25^. The present study analyzed soil fungal communities in a long-term cultivated wheat field and fields that converted to JA cultivation for one, two and three years, respectively. Our results demonstrated that soil fungal community composition and diversity differed among the wheat field and the first, second and third cropping of JA, which supported our hypotheses.

Generally, monocropping of the same crop is not a long-term sustainable practice because it can induce accumulation of plant species-specific soil-borne pathogens; while crop rotation can effectively prevent the accumulation of soil-borne pathogens^24^,^26^,^27^. Miseq sequencing showed that the long-term cultivated wheat field had higher relative abundances of potential pathogens of wheat, such as *Alternaria, Microdochium* and *Epicoccum* spp^28^. The relative abundances of *Gibberella* spp., a root pathogen of wheat^28^, and *Olpidium* spp., a soil-borne root-infecting pathogen and a vector of plant viruses^29^, were also higher in the first cropping of JA. However, these potential pathogens were lower in the second cropping of JA, indicating the cultivation of JA for two years can suppress certain pathogens of wheat. Fungal taxa with plant growth promoting, plant pathogen or insect antagonistic potentials such as *Cryptococcus*^30^, *Preussia*^31^, *Metarhizium*^32^, *Exophiala*^33^, *Trichosporon*^34^, *Clonostachys* spp.^35^ were enriched in the first cropping of JA, *Acremonium*^15^, *Ampelomyces*^36^ and *Penicillium* spp.^37^ were enriched in the second cropping of JA. Moreover, unique OTUs belonging to *Powellomyces*^38^ and *Preussia* spp.^31^ with plant-growth-promoting potentials appeared in the second and third cropping of JA, respectively. The qPCR analysis showed that the third cropping of JA had higher *Trichoderma* spp. abundance but lower *Fusarium* spp. abundance. These further indicated that rotation with JA may be adopted in wheat cultivation in order to suppress certain pathogens of wheat and stimulate fungal taxa beneficial to plants.

The relative abundances of *Mortierella* and *Stachybotrys* spp. increased in the long-term cultivated wheat field and *Lecythophora* and *Tetracladium* spp. increased in the first cropping of JA. These taxa are lignocellulose decomposer and are also involved in wheat residue decomposition^39^,^40^,^41^. Our results indicate that the soil fungal community can become compositionally adapted to utilize plant litter of a certain quality^42^. Other lignocellulose decomposers such as *Chaetomidium* spp.^43^ were higher in the second and third cropping of JA, while *Chaetomium*^44^ and *Hypocrea* spp.^45^ were higher in the third cropping of JA. Unique OTUs belonging to *Mortierella* spp. were detected in both the first and second cropping of JA. Plant litter and root exudates provide important carbon resources for soil microorganisms and changes in these plant-derived organic matters can affect soil microbial communities^8^,^9^. Wheat, belonging to the Poaceae family, is a herbaceous annual plant; while JA, belonging to the Asteraceae family, is a herbaceous perennial plant. It has been shown that plant litter chemistry differs among plant species^10^. For example, the chemical composition of both the leaf and root litter differed between wheat and sunflower (*H. annuus*), a close relative of JA^46^,^47^. Therefore, though no current data are available, the quality and quantity of root exudates and plant litter are supposed to be different between wheat and JA. The changes in these carbon resources induced by conversion from wheat to JA plantation may be responsible for the succession of decomposing soil biota observed in this study.

Compared with Miseq sequencing, fingerprinting methods are known to have lower resolution^48^, which was reflected by the lower total fugal community richness (number of visible bands) detected by PCR-DGGE than the community richness (OTU numbers) detected by Miseq sequencing. For *Fusarium* and *Trichoderma* spp. communities, the number of visible bands detected by PCR-DGGE were higher than OTU numbers detected by Miseq sequencing. This may be due to the fact that the amplicons analyzed by PCR-DGGE were longer (for partial *Fusarium Ef1α* gene, 450 bp^49^; for *Trichoderma* partial ITS region, 650 bp^50^) than the amplicons analyzed by Miseq sequencing (fungal partial ITS region, 280 bp^51^). However, caution also should be taken to interpret the diversity indices from PCR-DGGE results because individual microbial organisms can produce a number of different bands on a gel and a single band frequently comprises several different microbial species in the DGGE profiles^52^.

The decline in soil fungal community diversity observed in the third cropping of JA was consistent with previous studies showing that intensive agricultural management, especially successive monocropping, had an adverse effect on soil biodiversity^22^,^23^,^24^,^27^. The diversity of microbial communities and ecosystem functions are usually positively related^7^. For example, increasing microbial diversity can inhibit the invasion of pathogens^53^, promote plant productivity and other ecosystem functions such as litter decomposition, nutrient retention and cycling^7^,^25^. The third cropping of JA had the lowest soil Olsen P, indicating that the P mobilization decreased or P leaching increased. Our results suggested that JA cropping for three croppings may adversely affect belowground ecosystem functions. Therefore, effects of long-term JA cropping on soil functions need be further elucidated.

In our experiment, the first cropping of JA can be seen as a wheat-JA rotation treatment, which had more diverse plant litter than the wheat field, second and third cropping of JA. Our results showed that the first cropping of JA had higher soil SOC content, which supported the notion that increasing plant diversity can enhance the accumulation of soil carbon storage^54^. Mantel test showed that soil total fungal and *Fusarium* spp. community structures were significantly correlated to SOC and Olsen P, which consist with previous observations that soil carbon and P status are important drivers of changes in soil fungal communities^55^,^56^. Therefore, plantation conversion can affect soil fungal communities directly through providing different quantity and quality of organic matter, and indirectly through plant-mediated changes in soil properties.

In our field experiment, wheat and JA were flooding irrigated with groundwater when necessary for the crops. Wheat and JA fields would differ in the frequency of irrigation and amount of water supplied, which have been shown to influence soil microbial community activity and diversity^57^,^58^. Thus, irrigation may play some role in shaping soil fungal communities in our cropping system, which warrants further studies.

## Conclusions

Overall, our results demonstrated that conversion from wheat to JA plantation changed soil fungal community structure, composition and abundance, which were linked to changes in soil SOC and Olsen P content. Soil total fungal and *Fusarium* spp. communities differed not only between the wheat field and JA field but also among fields with differing JA-plantation years. Short-term conversion from wheat to JA plantation (one to three years) had positive effects on soil fungal communities by reducing certain potential plant fungal pathogens of wheat and promoting beneficial fungi. Future studies should focus on the influences of this land use conversion on the functions of soil microbial communities and its feedback effects on crop performances.

## Materials and Methods

### Field Experiment

The experiment site was located in a field of Mojiaquanwan village, Chengbei District, Xining, China (36°42′N, 101°45′E), which has been successively planted with wheat for more than 20 years. The annual precipitation was 378.3 mm, the mean annual temperature ranges from 2.6 to 5.3 °C. The soil was a castanozem soil (FAO/Unesco System of Soil Classification), contained 43% sand, 39% silt, 18% clay, organic matter, 2.03%; available N (NH_4_^+^ and NO_3_^−^), 69 mg kg^−1^; Olsen P, 65 mg kg^−1^; available K, 229 mg kg^−1^; and pH (1:2.5, w/v), 8.12.

The field experiment was conducted from April 2010 to October 2012. There were four types of plantation regimes in the experiment, namely, W, F, S and T (Table S7). W was the long-term cultivated wheat field. F, S and T were designed to successively cultivate JA for 1, 2 and 3 years, respectively. Briefly, in 2010, T was planted with JA, W, F and S were planted with wheat. In 2011, T and S were planted with JA, W and F were planted with wheat. In 2012, T, S and F were planted with JA, W was planted with wheat. There were three replicate plots (120-m long, 80-m wide) for each plantation regime, arranged in a randomized block design.

Though JA is a herbaceous perennial plant, it is usually grown as an annual crop in China by planting tubers. JA tubers (cv. Qingyu 2) were provided by Institute of Horticulture, Qinghai Academy of Agriculture and forestry Sciences, China. JA was planted on April 5 each year and harvested on October 25 each year. Within-row spacing was 40 cm and the row width was 60 cm. Wheat was broadcast seeded in early March and harvested in early September. There was one crop (wheat or JA) per year. After the harvest of JA and wheat, the fields were left fallow until next year. Both diammonium hydrogen phosphate and urea were applied at the rate of 300 kg ha^−1^ as basal fertilizer. Flooding irrigation with groundwater was performed when necessary. Weeds were manually removed once a month in May and June.

### Soil sampling

Bulk soil samples were collected on November 25, 2012, one month after JA harvest. Eight soil cores (5 cm diameter, 15 cm deep) were randomly collected from each plot to make a composite sample. Soil samples were sieved (2 mm) and stored at −70 °C. There were triplicate soil samples for each treatment and there were 12 soil samples in total.

### Soil chemical analysis

Soil pH was determined with 10 g soil in water suspensions at a soil/water ratio of 1:2.5 with a glass electrode. For soil Olsen P, inorganic N (NH_4_^+^-N and NO_3_^−^-N) and available K, soil was extracted with 0.5 M sodium bicarbonate, 2 M potassium chloride and 1 M ammonium acetate, respectively, and was determined with a continuous flow analyzer (San^++^, SKALAR, Netherlands). Soil SOC was analyzed by digesting 0.5 g soil with potassium dichromate and sulphuric acid, and titrating the residual potassium dichromate with ammonia ferrous sulphate.

### DNA extraction, Illumina Miseq sequencing and data processing

Total soil DNA was extracted with the PowerSoil DNA Isolation Kit (MO BIO Laboratories, Carlsbad, USA) according to the manufacturer’s instructions.

Soil total fungal community composition was analyzed with Illumina MiSeq sequencing as described before^59^. Briefly, primer sets ITS1F/ITS2^51^ were used to amplify the ITS1 region of fungal rRNA genes. Both the forward and reverse primers also had a 6-bp barcode unique to each sample, which were used to permit multiplexing of samples. DNA samples were amplified in triplicate on an ABI GeneAmp^®^ 9700 PCR System (ABI, MA, USA) in PCR reactions containing 2 μl 2.5 mM dNTPs, 0.4 μl FastPfu Polymerase (Transgen Biotech, Beijing, China), 0.4 μl 5× FastPfu Buffer, 0.8 μl each of the forward and reverse primers (5 μM), 0.2 μg bovine serum albumin, 10 ng soil DNA; sterile, deionized H_2_O was used to bring the total volume to 20 μl. Reactions were held at 95 °C for 3 min, with amplification proceeding for 35 cycles at 94 °C for 30 s, 55 °C for 30 s, and 72 °C for 45 s; a final extension of 10 min at 72 °C. The products of the triplicate PCR reactions of each soil sample were pooled and purified using the Agarose Gel DNA purification kit (TaKaRa, Dalian, China). Then, purified amplicons were quantified by a TBS-380 micro fluorometer with Picogreen reagent (Invitrogen, CA, USA), and mixed accordingly to achieve the equal concentration in the final mixture. The mixture was then paired-end sequenced (2 × 300) on an Illumina Miseq platform at Majorbio Bio-Pharm Technology Co., Ltd., Shanghai, China.

Raw sequence reads were de-multiplexed, quality-filtered, and processed using the QIIME^60^ as described before^61^. OTUs were delineated at 97% sequence similarity. Then, a representative sequence of each OTU was taxonomically classified with Unite database^62^. Chimeric sequences were identified and removed using USEARCH 6.1 in QIIME^60^. To correct for survey effort (number of sequences analyzed per sample), a subset of 34, 468 ITS sequences were randomly selected from each sample for further analysis. The data set was deposited in the NCBI- Sequence Read Archive with the submission Accession Number SRP083394 and SRP083434.

### PCR-DGGE analysis

Soil total fungal, Fusarium and *Trichoderma* spp. community structures were analyzed by PCR-DGGE method. The total fungal and *Trichoderma* spp. partial ITS regions, and *Fusarium* spp. *Ef1α* genes were nest amplified. Primer sets of ITS1F/ITS4^51^ and ITS1F-GC /ITS2^51^ were used for fungal ITS regions, ITS1F/ITS4*Tr*R and ITS*Tr*F-GC/ITS*Tr*R^50^ were used for *Trichoderma* ITS regions, EF-1/EF-2^63^ and Alfie1-GC/Alfie2^49^ were used for *Fusarium Ef1α* genes in the first and second round of PCR amplifications, respectively.

DGGE analysis of total fungal community was performed on an 8% (w/v) acrylamide gel with 20–60% denaturant gradient^24^, *Trichoderma* spp. community on a 6–9% (w/v) acrylamide gel with 30–60% denaturant gradient^50^, *Fusarium* spp. community on a 6% (w/v) acrylamide gel with 40–60% denaturant gradient^18^,^24^. The gel was run in a 1× TAE (Tris-acetate-EDTA) buffer for 12 h at 60 °C and 80 V with a DCode universal mutation detection system (Bio-Rad Lab, LA, USA). Then, the gel was stained in 1:3300 (v/v) GelRed (Biotium, USA) nucleic acid staining solution for 20 min and was photographed under 302 nm UV light.

### qPCR assay

Abundances of soil total fungal, *Fusarium* and *Trichoderma* spp. communities were determined by SYBR Green qPCR. The ITS regions of soil total fungal and *Trichoderma* spp. communities were quantified with ITS1F/ITS4^51^ and uTf/uTr 64, respective, as described before^24^,^65^. For *Fusarium* spp. community, the *Ef1α* gene was nested amplified with EF-1/EF-2^63^ and Alfie1/Alfie2^49^ as described by Yergeau *et al*.^49^. Care was made to ensure that the first-round PCR products were all in exponential amplification phase of the PCR^18^. The first round amplification of the *Ef1α* gene was conducted using the S1000^TM^ Thermal Cycler (Bio-Rad Lab, LA, USA) in a 50 μl reaction mixture that contained 25 μl of 2× Taq PCR MasterMix (Tiangen Biotech, Beijing, China), 0.2 μM of each primer, 5 ng of soil DNA. qPCR assays were conducted using the IQ5 real-time PCR system (Bio-Rad Lab, LA, USA) in a 20 μl reaction mixture containing 10 μl of 2× Real SYBR Mixture (Tiangen Biotech, Beijing, China), 0.2 μM of each primer, 2.5 ng of soil DNA for total fungal and *Trichoderma* spp. communities or 3 μl of 10-fold diluted first round PCR products for *Fusarium* spp. community. The PCR conditions were 94 °C for 5 min, 94 °C for 45 s, 57.5 °C for 45 s for fungal ITS region (or 55.5 °C for *Trichoderma* spp. ITS region; 53 °C and 67 °C for the first and second round amplification of *Fusarium* Ef1*α* gene), 72 °C for 90 s, 30 cycles for fungal ITS region (or 32 cycles for *Trichoderma* spp. ITS region; 35 and 30 cycles for the first and second round amplification of *Fusarium* Ef1*α* gene), 72 °C for 10 min. For soil total fungal and *Trichoderma* spp. communities, standard curves were created with 10-fold dilution series of plasmids containing the ITS regions from soil samples. The initial copy number of the target gene was calculated by comparing the threshold cycle values of each sample with the standard curve. The relative *Fusarium* spp. community abundance was calculated as described by Wakelin *et al*.^18^ and then, all treatments were compared with the wheat field and expressed as the percentage of the abundance in the wheat field. Sterile deionized water, instead of template, was used as the negative control. All amplifications were in triplicate. The specificity of the PCR products was checked by melting curve analysis and agarose gel electrophoresis.

### Statistical analysis

For the Illumina MiSeq sequencing data, Good’s coverage, Chao1, ACE, Shannon and Simpson diversity indices were generated using QIIME^60^. Microbial community composition (relative OTU abundance data) was analyzed using PCoA analysis based on weighted UniFrac distance matrix. Linear discriminant effect size (LEfSe) analysis was used to identify taxa that were significantly associated with each treatment using a threshold of 2.0 for logarithmic linear discriminant analysis (LDA) scores^66^. Heat map analysis was used to compare the relative abundances of the top 50 most abundant classified fungal genera among treatments. The shared and unique OTUs among treatments were counted, and their distributions shown in a Venn diagram.

Banding patterns of the DGGE profiles and CCA analysis were analyzed by the Quantity One software (version 4.5) and ‘R’^67^, respectively. The number of visible bands, Shannon and Evenness indices were calculated as described before^24^.

The data of soil chemical properties, diversity indices from the Miseq sequencing and DGGE analysis and soil microbial abundances from qPCR assays were analyzed by analysis of variance (ANOVA), and mean comparison between treatments was performed based on the Tukey’s honestly significant difference (HSD) test at the 0.05 probability level. The numerator and denominator degrees of freedom for the ANOVA results were 3 and 8, respectively. All data were tested for normality (Shapiro-Wilk’s test) and homogeneity of variances (Levene’s test). Data of total fungal and *Trichoderma* spp. community abundances were logarithmically transformed and data of the relative *Fusarium* spp. community abundances were subjected to an arcsine square root transformation.

ANOSIM, adonis and MRPP analyses were carried out to test the differences in microbial communities with the Bray-Curtis distance and 999 permutations. CCA analysis was conducted to determine which soil chemical properties are most frequently related to fungal community structure. Mantel test with a Monte Carlo simulation with 999 randomizations was used to assess the relationships between the Bray-Curtis distance of fungal community and Euclidean distances of soil chemical properties. The PCA, PCoA, ANOVA, ANOSIM, adonis, MRPP, CCA and Mantel test analyses were performed with the ‘vegan’ package, Heat map with ‘gplots’, and Venn diagram with the ‘VennDiagram’ in ‘R’^67^.

## Additional Information

**How to cite this article**: Zhou, X. *et al*. Conversion from long-term cultivated wheat field to Jerusalem artichoke plantation changed soil fungal communities. *Sci. Rep.* **7**, 41502; doi: 10.1038/srep41502 (2017).

**Publisher's note:** Springer Nature remains neutral with regard to jurisdictional claims in published maps and institutional affiliations.

## Supplementary Material

Supplementary Information

## Figures and Tables

**Table 1:** Summary statistics of Illumina MiSeq sequencing fungal ITS regions of soil samples from the wheat field (W), the first (F), second (S) and third (T) cropping of Jerusalem artichoke.

	Sequences	Number of OTUs	Chao1	ACE	Shannon index	Simpson index	Good’s coverage (%)
W	39958 ± 1344	460 ± 11^a^	529 ± 9^a^	513 ± 9^a,b^	4.11 ± 0.04^a^	0.0337 ± 0.0013^b^	99.74 ± 0.01
F	37699 ± 1632	501 ± 8^a^	577 ± 19^a^	565 ± 24^a^	4.07 ± 0.01^a^	0.0387 ± 0.0018^b^	99.71 ± 0.03
S	39861 ± 2475	479 ± 27^a^	587 ± 38^a^	563 ± 33^a^	3.73 ± 0.24^a^	0.0906 ± 0.0343^a,b^	99.67 ± 0.03
T	39091 ± 2979	322 ± 14^b^	417 ± 13^b^	411 ± 18^b^	3.09 ± 0.07^b^	0.1276 ± 0.0082^a^	99.74 ± 0.01

OTUs were delineated at 97% similarity. The Good’s coverage and diversity indices were calculated from 34,468 sequences per sample. Values (mean ± SE) with different letters are significantly different at the 0.05 probability level (Tukey’s HSD test).

**Table 2:** Soil chemical properties in the wheat field (W), the first (F), second (S) and third (T) cropping of Jerusalem artichoke.

	Soil pH	Soil SOC (g kg^−1^)	Inorganic N (mg kg^−1^)	Olsen P (mg kg^−1^)
W	8.12 ± 0.02^a^	11.77 ± 0.28^b^	68.65 ± 3.19^a^	65.44 ± 2.93^a^
F	8.23 ± 0.07^a^	16.44 ± 1.36^a^	53.79 ± 6.41^a^	73.29 ± 4.89^a^
S	8.25 ± 0.12^a^	12.85 ± 0.26^b^	68.34 ± 5.45^a^	75.80 ± 2.70^a^
T	8.31 ± 0.08^a^	11.09 ± 0.20^b^	73.22 ± 8.59^a^	50.22 ± 1.43^b^

Values (mean ± SE) with different letters are significantly different at the 0.05 probability level (Tukey’s HSD test).

## References

[b1] Vidotto F., Tesio F. & Ferrero A. Allelopathic effects of *Helianthus tuberosus* L. on germination and seedling growth of several crops and weeds. Biol Agric Hortic 26, 55–68 (2008).

[b2] Kaur N. & Gupta A. K. Applications of inulin and oligofructose in health and nutrition. J Biosciences 27, 703–714 (2002). 10.1007/BF02708379 12571376

[b3] Long X. H. . Seawater stress differentially affects germination, growth, photosynthesis, and ion concentration in genotypes of Jerusalem artichoke (*Helianthus tuberosus* L.). J Plant Growth Regul 29, 223–231 (2010).

[b4] Liu Z. X., Spiertz J. H. J., Sha J., Xue S. & Xie G. H. Growth and yield performance of Jerusalem artichoke clones in a semiarid region of China. Agron J 104, 1538–1546 (2012).

[b5] Zhang Z. . Economic benefits of Industrial planting Jerusalem artichoke in the coastal area of Jiangsu Province, China. Jiangsu J Agr Sci 43, 480–483 (2015).

[b6] Bardgett R. D. & van der Putten W. H. Belowground biodiversity and ecosystem functioning. Nature 515, 505–511 (2014). 25428498 10.1038/nature13855

[b7] Wagg C., Bender S. F., Widmer F. & van der Heijden M. G. A. Soil biodiversity and soil community composition determine ecosystem multifunctionality. Proc Natl Acad Sci 111, 5266–5270 (2014). 24639507 10.1073/pnas.1320054111 PMC3986181

[b8] Meier C. L. & Bowman W. D. Links between plant litter chemistry, species diversity, and below-ground ecosystem function. Proc Natl Acad Sci 105, 19780–19785 (2008). 19064910 10.1073/pnas.0805600105 PMC2604978

[b9] Wardle D. A. . Ecological linkages between aboveground and belowground biota. Science 304, 1629–1633 (2004). 15192218 10.1126/science.1094875

[b10] Birouste M., Kazakou E., Blanchard A. & Roumet C. Plant traits and decomposition: are the relationships for roots comparable to those for leaves? Ann Bot 109, 463–472 (2012). 22143881 10.1093/aob/mcr297 PMC3268542

[b11] Bais H. P., Park S. W., Weir T. L., Callaway R. M. & Vivanco J. M. How plants communicate using the underground information superhighway. Trends Plant Sci 9, 26–32 (2004). 14729216 10.1016/j.tplants.2003.11.008

[b12] Funnell-Harris D. L., Pedersen J. F. & Marx D. B. Effect of sorghum seedlings, and previous crop, on soil fluorescent *Pseudomonas* spp. Plant Soil 311, 173–187 (2008).

[b13] Bunemann E. K., Marschner P., Smernik R. J., Conyers M. & McNeill A. M. Soil organic phosphorus and microbial community composition as affected by 26 years of different management strategies. Biol Fert Soils 44, 717–726 (2008).

[b14] Ryan P. R., Dessaux Y., Thomashow L. S. & Weller D. M. Rhizosphere engineering and management for sustainable agriculture. Plant Soil 321, 363–383 (2009).

[b15] Vujanovic V., Mavragani D. & Hamel C. Fungal communities associated with durum wheat production system: A characterization by growth stage, plant organ and preceding crop. Crop Prot 37, 26–34 (2012).

[b16] Ma L. J. . Fusarium pathogenomics. Annu Rev Microbiol 67, 399–416 (2013). 24024636 10.1146/annurev-micro-092412-155650

[b17] Harman G. E., Howell C. R., Viterbo A., Chet I. & Lorito M. *Trichoderma* species-opportunistic, avirulent plant symbionts. Nat Rev Microbiol 2, 43–56 (2004). 15035008 10.1038/nrmicro797

[b18] Wakelin S. A., Warren R. A., Kong L. & Harvey P. R. Management factors affecting size and structure of soil *Fusarium* communities under irrigated maize in Australia. Appl Soil Ecol 39, 201–209 (2008).

[b19] Yergeau E. . Patterns of *Fusarium* community structure and abundance in relation to spatial, abiotic and biotic factors in soil. FEMS Microbiol Ecol 71, 34–42 (2010). 19780827 10.1111/j.1574-6941.2009.00777.x

[b20] Harman G. E. Overview of mechanisms and uses of *Trichoderma* spp. Phytopathology 96, 190–194 (2006). 18943924 10.1094/PHYTO-96-0190

[b21] Bever J. D., Platt T. G. & Morton E. R. Microbial population and community dynamics on plant roots and their feedbacks on plant communities. Annu Rev Microbiol 66, 265–283 (2012). 22726216 10.1146/annurev-micro-092611-150107 PMC3525954

[b22] Tiemann L. K., Grandy A. S., Atkinson E. E., Marin-Spiotta E. & McDaniel M. D. Crop rotational diversity enhances belowground communities and functions in an agroecosystem. Ecol Lett 18, 761–771 (2015). 26011743 10.1111/ele.12453

[b23] Tsiafouli M. A. . Intensive agriculture reduces soil biodiversity across Europe. Glob Change Biol 21, 973–985 (2015). 10.1111/gcb.12752 25242445

[b24] Zhou X. & Wu F. Dynamics of the diversity of fungal and *Fusarium* communities during continuous cropping of cucumber in the greenhouse. FEMS Microbiol Ecol 80, 469–478 (2012). 22273443 10.1111/j.1574-6941.2012.01312.x

[b25] van der Heijden M. G. . Mycorrhizal fungal diversity determines plant biodiversity, ecosystem variability and productivity. Nature 396, 69–72 (1998).

[b26] Cook R. J. Toward cropping systems that enhance productivity and sustainability. Proc Natl Acad Sci 103, 18389–18394 (2006). 17130454 10.1073/pnas.0605946103 PMC1693674

[b27] Zhou X., Yu G. & Wu F. Effects of intercropping cucumber with onion or garlic on soil enzyme activities, microbial communities and cucumber yield. Eur J Soil Biol 47, 279–287 (2011).

[b28] Lenc L., Kwasna H., Sadowski C. & Grabowski A. Microbiota in wheat roots, rhizosphere and soil in crops grown in organic and other production systems. J Phytopathol 163, 245–263 (2015).

[b29] Taheri A. E., Hamel C. & Gan Y. T. Pyrosequencing reveals the impact of foliar fungicide application to chickpea on root fungal communities of durum wheat in subsequent year. Fungal Ecol 15, 73–81 (2015).

[b30] Cloete K. J., Valentine A. J., Stander M. A., Blomerus L. M. & Botha A. Evidence of symbiosis between the soil yeast *Cryptococcus laurentii* and a sclerophyllous medicinal shrub, *Agathosma betulina* (Berg.) Pillans. Microbial Ecol 57, 624–632 (2009). 10.1007/s00248-008-9457-9 18958514

[b31] Kleczewski N. M., Bauer J. T., Bever J. D., Clay K. & Reynolds H. L. A survey of endophytic fungi of switchgrass (*Panicum virgatum*) in the Midwest, and their putative roles in plant growth. Fungal Ecol 5, 521–529 (2012).

[b32] Roberts D. W. & Leger R. J. S. *Metarhizium* spp., cosmopolitan insect-pathogenic fungi: Mycological aspects. Advan Appl Microbiol 54, 1–70 (2004). 15251275 10.1016/S0065-2164(04)54001-7

[b33] Khan A. L. . *Exophiala* sp. LHL08 association gives heat stress tolerance by avoiding oxidative damage to cucumber plants. Biol Fert Soils 48, 519–529 (2012).

[b34] El-Tarabily K. A. Suppression of *Rhizoctonia solani* diseases of sugar beet by antagonistic and plant growth-promoting yeasts. J Appl Microbiol 96, 69–75 (2004). 14678160 10.1046/j.1365-2672.2003.02043.x

[b35] Keyser C. A., Jensen B. & Meyling N. V. Dual effects of *Metarhizium* spp. and *Clonostachys rosea* against an insect and a seed-borne pathogen in wheat. Pest Manag Sci 72, 517–526 (2016). 25827357 10.1002/ps.4015

[b36] Kiss L., Russell J. C., Szentivanyi O., Xu X. & Jeffries P. Biology and biocontrol potential of *Ampelomyces* mycoparasites, natural antagonists of powdery mildew fungi. Biocontrol Sci Technol 14, 635–651 (2004).

[b37] Saleem M., Arshad M., Hussain S. & Bhatti A. S. Perspective of plant growth promoting rhizobacteria (PGPR) containing ACC deaminase in stress agriculture. J Ind Microbiol Biotechnol 34, 635–648 (2007). 17665234 10.1007/s10295-007-0240-6

[b38] Midgley D. J., Letcher P. M. & McGee P. A. Access to organic and insoluble sources of phosphorus varies among soil Chytridiomycota. Arch Microbiol 186, 211–217 (2006). 16868741 10.1007/s00203-006-0136-2

[b39] Bastian F., Bouziri L., Nicolardot B. & Ranjard L. Impact of wheat straw decomposition on successional patterns of soil microbial community structure. Soil Biol Biochem 41, 262–275 (2009).

[b40] Sherman C., Grishkan I., Barness G. & Steinberger Y. Fungal community-plant litter decomposition relationships along a climate gradient. Pedosphere 24, 437–449 (2014).

[b41] Ma A. . Ascomycota members dominate fungal communities during straw residue decomposition in arable soil. PLoS ONE 8, e66146 (2013). 23840414 10.1371/journal.pone.0066146 PMC3688710

[b42] Ayres E. . Home-field advantage accelerates leaf litter decomposition in forests. Soil Biol Biochem 41, 606–610 (2009).

[b43] Boonyuen N. . Fungal occurrence on sugarcane filter cake and bagasse isolated from sugar refineries in Thailand. Thai J Agr Sci 47, 77–86 (2014).

[b44] Longoni P. . Functional analysis of the degradation of cellulosic substrates by a *Chaetomium globosum* endophytic isolate. Appl Environ Microbiol 78, 3693–3705 (2012). 22389369 10.1128/AEM.00124-12 PMC3346360

[b45] Kubicek C. P., Mikus M., Schuster A., Schmoll M. & Seiboth B. Metabolic engineering strategies for the improvement of cellulase production by *Hypocrea jecorina*. Biotech Biofuels 2, 19 (2009). 10.1186/1754-6834-2-19 PMC2749017 19723296

[b46] Redin M. . Carbon mineralization in soil of roots from twenty crop species, as affected by their chemical composition and botanical family. Plant Soil 378, 205–214 (2014).

[b47] Garcia-Palacios P. . Side-effects of plant domestication: ecosystem impacts of changes in litter quality. New Phytol 198, 504–513 (2013). 23356416 10.1111/nph.12127

[b48] Hirsch P. R., Mauchline T. H. & Clark I. M. Culture-independent molecular techniques for soil microbial ecology. Soil Biol Biochem 42, 878–887 (2010).

[b49] Yergeau E., Filion M., Vujanovic V. & St-Arnaud M. A PCRdenaturing gradient gel electrophoresis approach to assess *Fusarium* diversity in asparagus. J Microbiol Meth 60, 143–154 (2005). 10.1016/j.mimet.2004.09.006 15590089

[b50] Meincke R. . Development of a molecular approach to describe the composition of *Trichoderma* communities. J Microbiol Meth 80, 63–69 (2010). 10.1016/j.mimet.2009.11.001 19896986

[b51] Gardes M. & Bruns T. D. ITS primers with enhanced specificity for basidiomycetes: application to the identification of mycorrhiza and rusts. Mol Ecol 2, 113–118 (1993). 8180733 10.1111/j.1365-294x.1993.tb00005.x

[b52] Hill G. T. . Methods for assessing the composition and diversity of soil microbial communities. Appl Soil Ecol 15, 25–36 (2000).

[b53] van Elsas J. D. . Microbial diversity determines the invasion of soil by a bacterial pathogen. Proc Natl Acad Sci 109, 1159–1164 (2012). 22232669 10.1073/pnas.1109326109 PMC3268289

[b54] Steinbeiss S. . Plant diversity positively affects short-term soil carbon storage in experimental grasslands. Glob Change Biol 14, 2937–2949 (2008).

[b55] Lauber C. L., Strickland M. S., Bradford M. A. & Fierer N. The influence of soil properties on the structure of bacterial and fungal communities across land-use types. Soil Biol Biochem 40, 2407–2415 (2008).

[b56] Liu J. J. . Soil carbon content drives the biogeographical distribution of fungal communities in the black soil zone of northeast China. Soil Biol Biochem 83, 29–39 (2015).

[b57] Williams M. A. & Rice C. W. Seven years of enhanced water availability influences the physiological, structural, and functional attributes of a soil microbial community. Appl Soil Ecol 35, 535–545 (2007).

[b58] Bapiri A., Baath E. & Rousk J. Drying-rewetting cycles affect fungal and bacterial growth differently in an arable soil. Microbial Ecol 60, 419–428 (2010). 10.1007/s00248-010-9723-5 20635180

[b59] Crowther T. W. . Predicting the responsiveness of soil biodiversity to deforestation: a cross-biome study. Glob Change Biol 20, 2983–2994 (2014). 10.1111/gcb.12565 24692253

[b60] Caporaso J. G. . QIIME allows analysis of high-throughput community sequencing data. Nat Methods 7, 335–336 (2010). 20383131 10.1038/nmeth.f.303 PMC3156573

[b61] Zhong Y., Yan W. & Shangguan Z. Impact of long-term N additions upon coupling between soil microbial community structure and activity, and nutrient-use efficiencies. Soil Biol Biochem 91, 151–159 (2015).

[b62] Kõljalg U. . Towards a unified paradigm for sequence-based identification of fungi. Mol Ecol 22, 5271–5277 (2013). 24112409 10.1111/mec.12481

[b63] O’Donnell K., Kistler H. C., Cigelnik E. & Ploetz R. C. Multiple evolutionary origins of the fungus causing Panama-disease of banana: concordant evidence from nuclear and mitochondrial gene genealogies. Proc Natl Acad Sci 95, 2044–2049 (1998). 9482835 10.1073/pnas.95.5.2044 PMC19243

[b64] Hagn A., Wallisch S., Radl V., Charles Munch J. & Schloter M. A new cultivation independent approach to detect and monitor common *Trichoderma* species in soils. J Microbiol Method 69, 86–92 (2007). 10.1016/j.mimet.2006.12.004 17234287

[b65] Drigo B., van Veen J. A. & Kowalchuk G. A. Specific rhizosphere bacterial and fungal groups respond differently to elevated atmospheric CO_2_. ISME J 3, 1204–1217 (2009). 19536195 10.1038/ismej.2009.65

[b66] Segata N. . Metagenomic biomarker discovery and explanation. Genome Biol 12, R60 (2011). 21702898 10.1186/gb-2011-12-6-r60 PMC3218848

[b67] Core Team R. R: A language and environment for statistical computing. R Foundation for Statistical Computing, Vienna, Austria. http://www.R-project.org/ (2013).

