# Supplementary material for: Conversion from long-term cultivated wheat field to Jerusalem artichoke plantation changed soil fungal communities
Source: Sci Rep. 2017 Jan 30;7:41502. doi: 10.1038/srep41502 (PMC5278349; doi:10.1038/srep41502)
Supplement: Supplementary Information [file srep41502-s1.doc]

**Conversion from long-term cultivated wheat field to Jerusalem artichoke plantation changed soil fungal communities**

Xingang Zhou^1, 2^, Jianhui Zhang^1^, Danmei Gao^1^, Huan Gao^1^, Meiyu Guo^1^, Li Li^3^, Mengliang Zhao^3^, Fengzhi Wu^1^

^1^Department of Horticulture, Northeast Agricultural University, Harbin, China

^2^Key Laboratory of Biology and Genetic Improvement of Horticultural Crops (Northeast Region), Ministry of Agriculture, Harbin, China

^3^Institute of Horticulture, Qinghai Academy of Agriculture and Forestry Sciences, Xining, China

Correspondence and requests for materials should be addressed to F.W. (fzwuneau@yahoo.com).

Figure captions:

**Figure S1.** Rarefaction curves (a) and the Shannon-Wiener index (b) of soil fungal communities based on observed OTUs at 97% similarity in the wheat field (W), the first (F), second (S) and third (T) cropping of Jerusalem artichoke.

**Figure S2.** Cladograms, generated from LEfSe analysis, represent the polygenetic distribution of soil fungal taxa enriched in the wheat field (W, in purple), the first (F, in red), second (S, in green) and third (T, in blue) cropping of Jerusalem artichoke. Yellow circles represent non-significant differences in abundance between treatment groups for that particular taxa. Labels are shown of the phylum, class and order levels.

**Figure S3.** DGGE profile of soil total fungal (a), *Fusarium* (b) and *Trichoderma* spp. (c) communities in the wheat field (W), the first (F), second (S) and third (T) cropping of Jerusalem artichoke.

**Table S1.** Relative abundances (%) of the top 50 most abundant classified fungal genera in the wheat field (W), the first (F), second (S) and third (T) cropping of Jerusalem artichoke. Values (mean±SE) with different letters are significantly different at the 0.05 probability level (Kruskal Wallis test).

|  | W | F | S | T |  |  | W | F | S | T |
| --- | --- | --- | --- | --- | --- | --- | --- | --- | --- | --- |
| *Mortierella* | 29.92±1.86 a | 22.32±3.61 ab | 13.15±1.90 bc | 6.99±0.59 c |  | *Ampelomyces* | 0.03±0.01 b | 0.30±0.02 b | 1.07±0.21 a | 0.12±0.03 b |
| *Chaetomium* | 2.08±0.27 b | 2.17±0.33 b | 0.84±0.11 b | 24.59±2.47 a |  | *Volutella* | 0.18±0.02 ab | 0.55±0.13 ab | 0.72±0.20 a | 0.06±0.01 b |
| *Tetracladium* | 2.95±0.21 c | 8.20±0.28 a | 4.34±0.37 b | 0.90±0.08 d |  | *Cladorrhinum* | 0.03±0.01 a | 1.29±0.63 a | 0.00±0.00 a | 0.00±0.00 a |
| *Humicola* | 4.68±0.44 a | 0.27±0.02 b | 3.45±0.48 a | 1.71±0.24 b |  | *Exophiala* | 0.36±0.09 b | 0.63±0.00 a | 0.17±0.06 bc | 0.08±0.02 c |
| *Cryptococcus* | 1.95±0.32 b | 4.06±0.53 a | 1.63±0.42 b | 1.51±0.07 b |  | *Podospora* | 0.24±0.08 a | 0.14±0.01 a | 0.23±0.04 a | 0.40±0.19 a |
| *Kernia* | 0.17±0.01 a | 6.14±3.16 a | 0.01±0.00 a | 0.02±0.00 a |  | *Doratomyces* | 0.16±0.02 a | 0.17±0.04 a | 0.27±0.07 a | 0.34±0.05 a |
| *Guehomyces* | 1.92±0.21 a | 1.57±0.08 a | 1.29±0.30 ab | 0.59±0.07 b |  | *Microdochium* | 0.40±0.01 a | 0.13±0.01 b | 0.09±0.02 b | 0.18±0.07 b |
| *Leptosphaeria* | 1.49±0.58 a | 1.38±0.03 a | 1.12±0.19 a | 0.77±0.07 a |  | *Stachybotrys* | 0.36±0.04 a | 0.14±0.02 b | 0.16±0.04 b | 0.07±0.01 b |
| *Pseudaleuria* | 0.40±0.05 b | 0.00±0.00 b | 0.00±0.00 b | 4.12±0.70 a |  | *Parasola* | 0.05±0.01 bc | 0.01±0.01 c | 0.31±0.10 a | 0.28±0.02 ab |
| *Preussia* | 0.82±0.07 bc | 1.91±0.07 a | 1.02±0.04 b | 0.59±0.02 c |  | *Penicillium* | 0.06±0.02 b | 0.14±0.01 b | 0.41±0.11 a | 0.04±0.01 b |
| *Psathyrella* | 0.01±0.00 a | 0.04±0.00 a | 3.69±1.98 a | 0.01±0.01 a |  | *Fusarium* | 0.17±0.02 a | 0.25±0.11 a | 0.15±0.02 a | 0.06±0.02 a |
| *Gibberella* | 0.41±0.04 b | 2.17±0.14 a | 0.48±0.12 b | 0.34±0.02 b |  | *Trichosporon* | 0.12±0.04 b | 0.40±0.10 a | 0.05±0.02 b | 0.03±0.01 b |
| *Cylindrocarpon* | 1.38±0.02 a | 0.62±0.01 b | 0.54±0.04 b | 0.67±0.06 b |  | *Clonostachys* | 0.15±0.03 b | 0.30±0.04 a | 0.08±0.03 b | 0.05±0.01 b |
| *Acremonium* | 0.83±0.10 b | 0.30±0.01 b | 1.66±0.28 a | 0.21±0.03 b |  | *Paraphoma* | 0.05±0.01 c | 0.19±0.04 ab | 0.23±0.01 a | 0.09±0.02 bc |
| *Alternaria* | 2.54±0.16 a | 0.11±0.04 b | 0.22±0.05 b | 0.04±0.02 b |  | *Davidiella* | 0.38±0.08 a | 0.03±0.01 b | 0.12±0.02 b | 0.01±0.00 b |
| *Cephaliophora* | 0.25±0.01 b | 2.01±0.07 a | 0.20±0.03 b | 0.35±0.05 b |  | *Chaetomidium* | 0.07±0.00 b | 0.08±0.00 b | 0.27±0.05 a | 0.11±0.02 b |
| *Ilyonectria* | 0.55±0.02 b | 0.13±0.02 c | 0.90±0.02 a | 0.86±0.13 a |  | *Cladosporium* | 0.25±0.04 a | 0.06±0.00 b | 0.16±0.03 ab | 0.06±0.02 b |
| *Wardomyces* | 0.60±0.14 ab | 0.53±0.02 ab | 0.90±0.18 a | 0.14±0.01 b |  | *Paecilomyces* | 0.16±0.02 a | 0.17±0.02 a | 0.05±0.02 a | 0.14±0.05 a |
| *Nectria* | 0.42±0.02 ab | 0.51±0.05 ab | 0.68±0.06 a | 0.40±0.08 b |  | *Olpidium* | 0.15±0.02 b | 0.26±0.01 a | 0.04±0.01 c | 0.04±0.02 c |
| *Cephalosporium* | 0.16±0.01 b | 0.47±0.08 ab | 0.58±0.07 a | 0.68±0.11 a |  | *Pseudeurotium* | 0.21±0.02 a | 0.10±0.01 b | 0.10±0.01 b | 0.05±0.00 b |
| *Dendryphion* | 0.58±0.01 a | 0.65±0.07 a | 0.51±0.13 a | 0.13±0.03 b |  | *Trichoderma*/*Hypocrea* | 0.01±0.00 b | 0.02±0.00 b | 0.05±0.01 b | 0.32±0.02 a |
| *Coprinellus* | 0.89±0.55 a | 0.01±0.00 a | 0.23±0.10 a | 0.69±0.41 a |  | *Sporidiobolus* | 0.18±0.06 a | 0.13±0.01 ab | 0.03±0.01 b | 0.04±0.02 ab |
| *Metarhizium* | 0.07±0.03 b | 1.68±0.13 a | 0.02±0.00 b | 0.00±0.00 b |  | *Epicoccum* | 0.23±0.02 a | 0.02±0.01 b | 0.04±0.02 b | 0.01±0.00 b |
| *Typhula* | 0.20±0.13 a | 1.47±0.91 a | 0.01±0.01 a | 0.00±0.00 a |  | *Lecythophora* | 0.06±0.01 b | 0.18±0.01 a | 0.00±0.00 c | 0.03±0.01 bc |
| *Pseudogymnoascus* | 0.33±0.05 a | 0.13±0.04 a | 0.80±0.30 a | 0.36±0.10 a |  | *Mrakia* | 0.00±0.00 b | 0.04±0.00 ab | 0.11±0.03 a | 0.06±0.02 ab |

**Table S2.** Dissimilarity analysis of soil total fungal and *Fusarium* spp. communities with analysis of similarities (ANOSIM), non-parametric multivariate ANOVA (adonis), and multiple response permutation procedure (MRPP).

|  | ANOSIM | |  | adonis | | |  | MRPP | | |
| --- | --- | --- | --- | --- | --- | --- | --- | --- | --- | --- |
|  | R | P |  | F | R^2^ | P |  | Delta (*δ*) | Effect size (A) | P |
| Fungal community based  on Miseq sequencing | 1 | 0.001 |  | 17.889 | 0.870 | 0.001 |  | 0.204 | 0.581 | 0.001 |
| Fungal community based  on PCR-DGGE | 1 | 0.001 |  | 25.097 | 0.904 | 0.001 |  | 0.122 | 0.640 | 0.001 |
| *Fusarium* spp. community based  on PCR-DGGE | 1 | 0.001 |  | 66.943 | 0.962 | 0.001 |  | 0.061 | 0.774 | 0.001 |

**Table S3.** Sequence frequencies (%) of OTUs all shared and unique to the wheat field (W), the first (F), second (S) and third (T) cropping of Jerusalem artichoke at the class level.

|  | All shared OTUs | Unique OTUs | | | |
| --- | --- | --- | --- | --- | --- |
|  |  | W | F | S | T |
| Agaricomycetes | 0.71 | 5.50 | 10.36 | 0.81 | 5.08 |
| Archaeorhizomycetes |  |  | 0.58 |  |  |
| Ascomycota unclassified | 15.56 | 20.59 | 10.70 | 16.19 | 1.69 |
| Basidiomycota unclassified | 0.02 | 0.38 | 2.30 | 0.27 | 0.56 |
| Chytridiomycetes | 0.16 |  |  | 7.56 | 2.26 |
| Dothideomycetes | 6.65 | 3.32 | 4.83 | 6.61 | 53.11 |
| Eurotiomycetes | 0.71 | 1.02 | 5.18 | 3.24 | 1.69 |
| Exobasidiomycetes |  |  |  | 0.40 |  |
| Fungi unclassified | 0.44 | 48.59 | 13.69 | 19.03 | 5.08 |
| Glomeromycetes |  |  | 1.15 | 0.40 | 1.13 |
| Leotiomycetes | 9.73 | 1.28 | 14.61 | 3.10 |  |
| Microbotryomycetes | 0.10 | 0.64 |  | 1.21 |  |
| Pezizomycetes | 4.50 | 0.26 | 1.50 | 0.00 | 7.91 |
| Saccharomycetes |  |  | 0.35 | 0.27 |  |
| Sordariomycetes | 38.42 | 17.52 | 17.38 | 26.72 | 14.12 |
| Tremellomycetes | 4.26 | 0.38 | 1.50 | 0.67 |  |
| Wallemiomycetes | 0.01 |  |  |  |  |
| Zygomycetes | 18.73 | 0.51 | 15.88 | 13.50 | 7.34 |

**Table S4.** Number of sequences belong to *Fusarium* and *Trichoderma*/*Hypocrea* spp. as determined by Illumina MiSeq sequencing in the wheat field (W), the first (F), second (S) and third (T) cropping of Jerusalem artichoke. Values (mean±SE) with letters are significantly different at the 0.05 probability level (Tukey's HSD test).

|  | OTU ID | W | F | S | T |
| --- | --- | --- | --- | --- | --- |
| *Fusarium* spp. | OTU504 | 41±1 a | 26±6 ab | 42±7 a | 14±3 b |
|  | OTU666 | 0 a | 1±0 a | 0 a | 0 a |
|  | OTU721 | 18±7 a | 58±35 a | 6±3 a | 5±4 a |
|  | OTU999 | 0 a | 1±1 a | 3±2 a | 0 a |
| *Trichoderma*/*Hypocrea* spp. | OTU703 | 3±1 b | 8±1 b | 16±3 b | 110±8 a |

**Table S5.** Number of visible bands (*S*), Shannon diversity index (*H*) and Evenness index (*E*) based on DGGE analysis of soil total fungal and *Fusarium* spp. communities from the wheat field (W), the first (F), second (S) and third (T) cropping of Jerusalem artichoke. Values (mean±SE) with different letters are significantly different at the 0.05 probability level (Tukey's HSD test).

|  | Fungal community | | |  | *Fusarium* spp. community | | |
| --- | --- | --- | --- | --- | --- | --- | --- |
|  | *S* | *H* | *E* |  | *S* | *H* | *E* |
| W | 31.33±2.08 bc | 3.32±0.06 a | 0.86±0.02 a |  | 21.67±0.58 a | 2.96±0.04 a | 0.88±0.01 a |
| F | 35.33±0.58 a | 3.36±0.01 a | 0.87±0.00 a |  | 21.67±0.58 a | 2.95±0.03 a | 0.88±0.01 a |
| S | 34.33±1.53 ab | 3.32±0.04 a | 0.86±0.01 a |  | 17.67±0.58 b | 2.75±0.05 b | 0.82±0.02 b |
| T | 30.00±1.00 c | 3.29±0.02 a | 0.85±0.01 a |  | 12.00±0.00 c | 2.33±0.01 c | 0.69±0.00 c |

**Table S6.** The standardized Mantel statistics (r) from Mantel tests of correlations between dissimilarity matrices of soil fungal community structures and soil properties. Values in bold indicate significant correlations (P<0.05).

|  | pH | |  | Soil organic carbon | |  | Inorganic N | |  | Olsen P | |
| --- | --- | --- | --- | --- | --- | --- | --- | --- | --- | --- | --- |
|  | r | P |  | r | P |  | r | P |  | r | P |
| Fungal community based  on Miseq sequencing | -0.072 | 0.727 |  | **0.410** | **<0.001** |  | 0.126 | 0.114 |  | **0.211** | **0.048** |
| Fungal community based  on PCR-DGGE | -0.126 | 0.886 |  | **0.622** | **<0.001** |  | 0.224 | 0.052 |  | **0.246** | **0.040** |
| *Fusarium* spp. community based  on PCR-DGGE | -0.010 | 0.474 |  | **0.163** | **0.036** |  | -0.031 | 0.598 |  | **0.371** | **0.006** |

**Table S7.** Experiment setup of the field experiment illustrating crop species, wheat or Jerusalem artichoke (JA), cultivated in each plantation regime from 2010 to 2012.

|  | 2010 | 2012 | 2013 |
| --- | --- | --- | --- |
| W | Wheat | Wheat | Wheat |
| F | Wheat | Wheat | JA |
| S | Wheat | JA | JA |
| T | JA | JA | JA |

**Figure S1.**


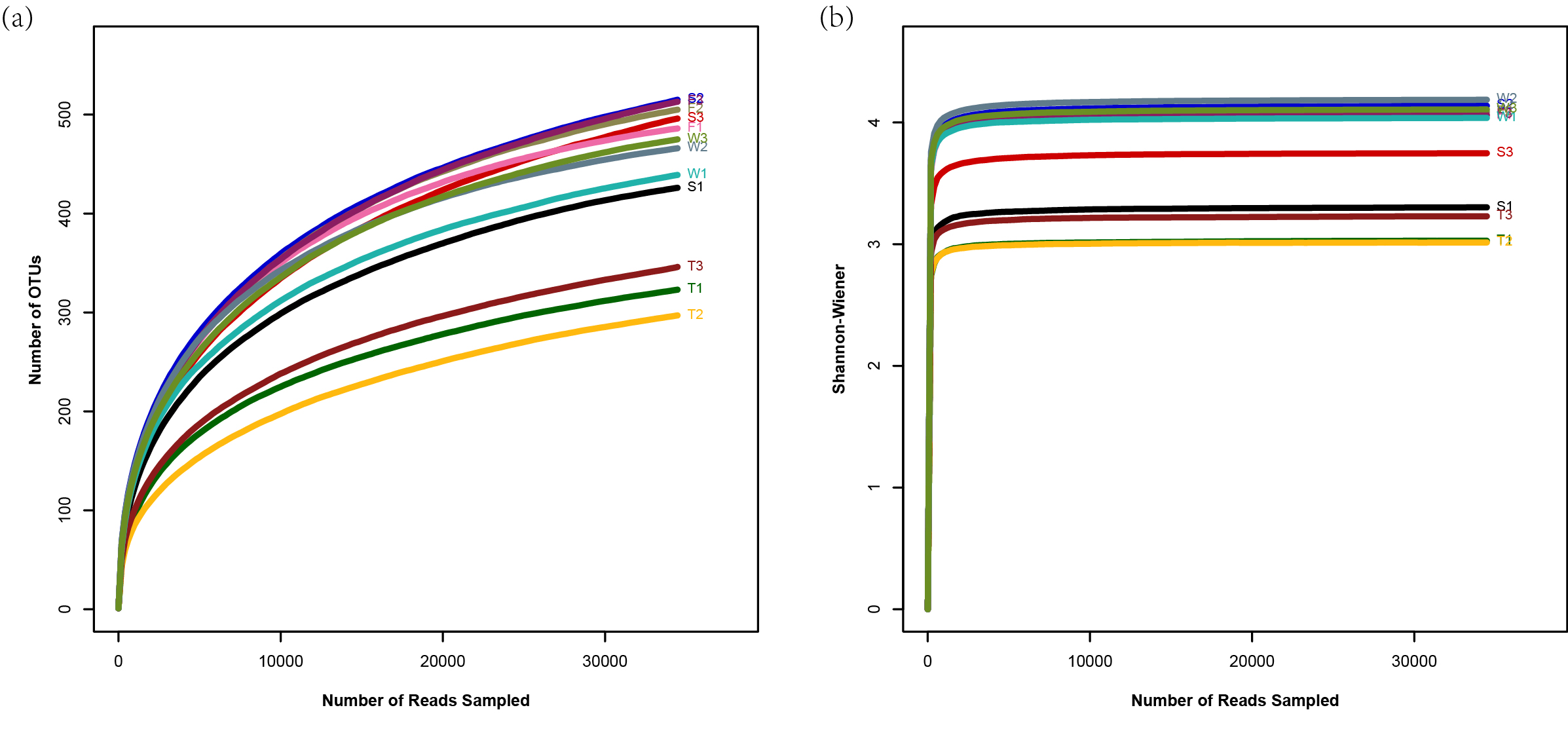


**Figure S2.**


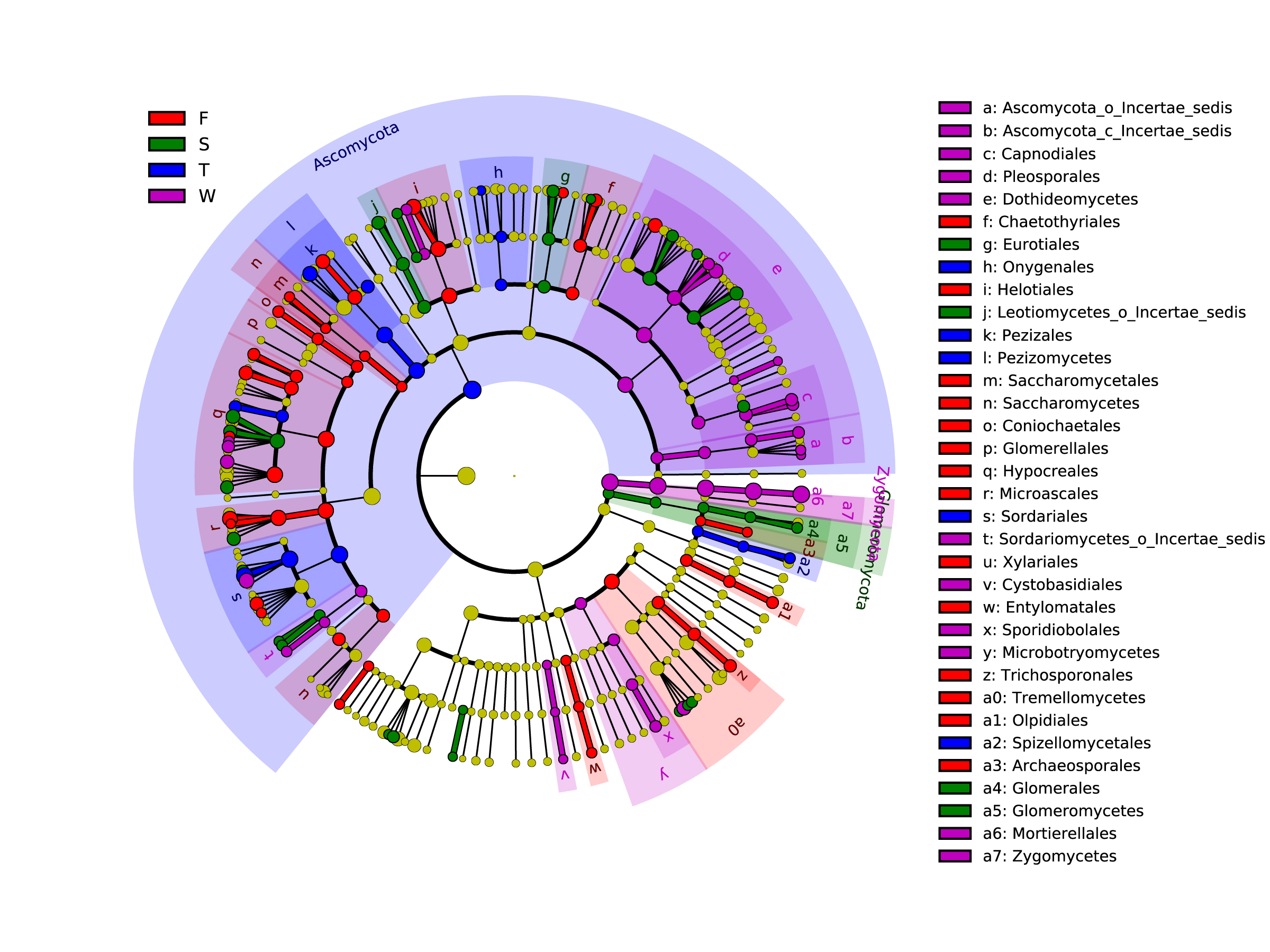


**Figure S3.**
